# A Novel CXCR4 Targeting Protein SDF-1/54 as an HIV-1 Entry Inhibitor

**DOI:** 10.3390/v11090874

**Published:** 2019-09-18

**Authors:** Suiyi Tan, Wenjuan Li, Zhaofeng Li, Yujing Li, Jiangyan Luo, Liangzhentian Yu, Jie Yang, Mengjie Qiu, Hongyan Cheng, Wei Xu, Shibo Jiang, Lu Lu, Shuwen Liu, Weifeng Ma

**Affiliations:** 1 Guangdong Provincial Key Laboratory of New Drug Screening, School of Pharmaceutical Sciences, Southern Medical University, Guangzhou 510515, China; 2 Department of Microbiology, School of Public Health, Southern Medical University, Guangzhou 510515, China; 3 Key Laboratory of Medical Molecular Virology of Ministries of Education & Health, Shanghai Medical College and Institute of Medical Microbiology, Fudan University, Shanghai 200032, China

**Keywords:** SDF-1/54, HIV-1, CXCR4, entry inhibitor

## Abstract

CXC chemokine receptor 4 (CXCR4) is a co-receptor for HIV-1 entry into target cells. Its natural ligand, the chemokine SDF-1, inhibits viral entry mediated by this receptor. However, the broad expression pattern of CXCR4 and its critical roles in various physiological and pathological processes indicate that the direct application of SDF-1 as an entry inhibitor might have severe consequences. Previously, we constructed an effective SDF-1 mutant, SDF-1/54, by deleting the α-helix of the C-terminal functional region of SDF-1. Of note, SDF-1/54 shows remarkable decreased chemotoxic ability, but maintains a similar binding affinity to CXCR4, suggesting SDF-1/54 might better serve as a CXCR4 inhibitor. Here, we found that SDF-1/54 exhibited potent antiviral activity against various X4 HIV-1 strains, including the infectious clone HIV-1 NL4-3, laboratory-adapted strain HIV-1 IIIB, clinical isolates and even drug-resistant strains. By using time-of-addition assay, non-infectious and infectious cell–cell fusion assay and CXCR4 internalization assay, we demonstrated SDF-1/54 is an HIV-1 entry inhibitor. A combination of SDF-1/54 with several antiretroviral drugs exhibited potent synergistic anti-HIV-1 activity. Moreover, SDF-1/54 was stable and its anti-HIV-1 activity was not significantly affected by the presence of seminal fluid, vaginal fluid simulant and human serum albumin. SDF-1/54 showed limited in vitro cytotoxicity to lymphocytes and vaginal epithelial cells. Based on these findings, SDF-1/54 could have a therapeutic potential as an HIV-1 entry inhibitor.

## 1. Introduction

Nearly 37 million people are currently living with human immunodeficiency virus type I (HIV-1) and approximately 2.1 million new infections per year continue to fuel the HIV-1 pandemic [1]. Current anti-retroviral (ARV) therapies for HIV/AIDS containing three or more drugs from at least two different ARV categories are usually recommended for use in combination to better suppress virus replication and to minimize drug resistance. Due to the long-term toxicity, high cost, drug-resistance and cross-resistance, HIV-1-infected patients might not be capable to keep a lifelong ARV treatment. Therefore, there is an urgent need to develop new, efficient, safe and cheap anti-HIV-1 drugs.

The virus–cell fusion process is an attractive target for the development of drugs to prevent HIV-1 infection of its target cells. These entry inhibitors can be subdivided into three different classes, including (i) adhesion inhibitors, targeting the gp120-CD4 binding; (ii) co-receptor antagonists targeting CCR5 or CXCR4 and (iii) fusion inhibitor. Currently, only T-20 targeting gp41 [2] and maraviroc (MVC), a CCR5 inhibitor [3], have been approved for clinical use by the FDA, but there is no entry inhibitor targeting CXCR4 clinically. Although R5-utilizing HIV-1 strains are often associated with the initial infection phase of the virus [4], as the infection progresses toward AIDS, X4R5 dual-tropic HIV-1 or X4-monotropic HIV-1 emerge [5]. The appearance of these forms is highly associated with accelerated disease progression and the decline of CD4^+^ T cells [5]. So, it is expected that the development of CXCR4 inhibitors will contribute to the treatment of AIDS.

Several peptides (such as T140) and macrocycles (such as AMD3100) were initially reported to be inhibitors targeting CXCR4 [6]. However, those antagonists can effectively inhibit the entry of X4-tropic HIV-1 strains, but not dual-tropic and R5-tropic HIV-1 strains. Moreover, some of those inhibitors have remarkable toxicity and side effects. For example, AMD3100 can inhibit the infection of HIV-1, but it was forced to stop research and development in phase II clinical trials due to its side effects on abnormal heart rate. AMD3100 has been approved as a CXCR4 antagonist by the United States FDA and only for stem cell mobilization in combination with G-CSF in patients with non-Hodgkin lymphoma and multiple myeloma [7]. It happens that there is a similar case, AMD070, a mutant of AMD3100, which not only has good anti-HIV-1 activity against X4-tropic virus, but also against R5 and dual-tropic HIV-1 strains. However, in long-term animal experiments, it has been found that AMD070 can lead to abnormal liver histomorphology and its clinical research has also been terminated by the United States FDA [8]. Berg C et al. found that the compound CXCR4-F292A7.43 not only inhibited the membrane fusion of HIV-1, but also hardly destroyed the signal transduction mediated by CXCR4, which made it possible to use small molecular inhibitors targeting CXCR4 to treat HIV-1 [9].

Unlike other chemokine receptors, CXCR4 has only one ligand, CXCL12 (CXC chemokine ligand-12), also known as stromal cell-derived factor-1 (SDF-1) [10]. SDF-1 is a CXC chemokine with a molecular weight of about 8–14 kD. It contains two cysteines and is classified as a member of the CXC subfamily. Chemokines have three tertiary folds, including the N-terminal region, a region that follows CXC or CC, three antiparallel β-strands and a C-terminal α-helix [11,12]. The N- terminal region contains the binding sites to the receptor [13]. Now, the molecular mechanism of recognition and activation of SDF-1 and CXCR4 has not been completely clarified by researchers [14]. It is reported that the conformation of SDF-1 C-terminal α-helix was related to its biological activity [13,14,15]. Mutants of SDF-1 might be used as inhibitors of CXCR4 to inhibit HIV-1 infection [7].

In view of this, our team deleted the α-helix of the C-terminal functional region of SDF-1 and obtained an efficient CXCR4-specific antagonist SDF-1/54. Previous experimental results showed that SDF-1/54 retained the ability to bind to CXCR4. The difference is that the binding to CXCR4 cannot induce the migration of tumor cells, nor the activation of downstream signal pathways. Thus, SDF-1/54 might not induce the normal physiological function of native SDF-1 [16]. These results suggest SDF-1/54 may be a safe and effective HIV-1 entry inhibitor. We hypothesized that SDF-1/54 could inhibit the entry of HIV-1 into host cells by inducing internalization of CXCR4 and blocking the binding of gp120 to CXCR4. In this paper, we aimed at investigating the anti-HIV-1 effect and the mechanism of SDF-1/54.

## 2. Materials and Methods

### 2.1. Reagents

Zidovudine (AZT), Raltegravir, Tenofovir and Nevirapine were bought from TargetMol (USA). AMD3100 and Histopaque-1077 were obtained from Sigma (USA). Anti-HIV p24 antibody (ab63913) was purchased from Abcam (UK). PE-conjugated mouse anti-human CXCR4 (clone12G5) antibody was purchased from R&D Systems (USA). Plasmids of CXCR4-tropic NL4-3 and CCR5-tropic SF162 infectious clone were kindly provided by Jan Münch of Ulm University. HIV-1 IIIB, 183-12H-5C, MT-2 cells, HEK293T cells, CHO-WT cells, TZM-b1 cells, H9/HIV-1 IIIB cells and Hela cells, were obtained from NIH AIDS Reference Reagent Program. CEMx174 5.25M7 cells were kindly provided by C. Cheng-Mayer. Ect/E6E7 cells, HEC-1-A cells and HEC-1-B cells were bought from ATCC. MT-2 cells and CEMx174 5.25M7 cells were maintained in RPMI 1640 medium supplemented with 10% fetal bovine serum (FBS) and 1% Penicillin/streptomycin at 37 °C and 5% CO_2_. HEK293T cells, CHO-WT cells and TZM-b1 cells were grown in Dulbecco’s modified Eagle’s medium (DMEM) supplemented with 10% FBS and 1% penicillin/streptomycin at 37 °C and 5% CO_2_. In addition, Hela cells, Ect/E6E7 cells, HEC-1-A cells and HEC-1-B cells were maintained in Minimum Essential Medium (MEM) supplemented with 1% penicillin/streptomycin and 10% FBS. Peripheral blood mononuclear cells (PBMC) were isolated from the blood of healthy donors by standard density gradient centrifugation using Histopaque-1077. The cells were plated in 75-cm^2^ flasks and incubated at 37 °C for 2 h. the non-adherent cells were collected and resuspended at 5 × 10^6^ in 20 mL of RPMI 1640 medium containing 10% FBS, 5 μg/mL phytohemagglutinin (PHA) and 100U/mL interleukin-2 (IL-2), followed by incubation at 37 °C for 3 days. SDF-1/54 protein was prepared by genetic engineering method as described elsewhere [16].

### 2.2. Preparation of HIV-1

Proviral plasmid DNA clones pNL4-3 and pSF162 were propagated as previously described [17,18]. Briefly, each plasmid was transfected into HEK293T cells in a six-well plate by using the calcium phosphate method. Supernatants containing the viruses were harvested 72 h post-transfection. HIV-1 IIIB, primary clinical isolates and the resistant strains were propagated in MT-2 cells or PBMC. Briefly, cells were infected with viruses, after a 4-day or 7-day incubation, cell-free supernatant was harvested and stored at −80 °C.

### 2.3. Single-Cycle Viral Infection

The inhibitory activities of the tested anti-viral agents against HIV-1 in TZM-bl cells were determined as described elsewhere [19]. Briefly, one day prior to infection, 100 μL of TZM-bl cells were added to each well of a 96-well plate at a density of 1 × 10^5^/mL. Plates were incubated overnight at 37 °C. On the day of infection, cells were pre-treated with serially diluted inhibitors in 50 μL for 15 min at 37 °C. Then the cells were infected with HIV-1 at 100 TCID_50_ (50% tissue culture infective dose) in a total volume of 200 μL. At 48 h, the cells were washed and lysed by lysing buffer. Aliquots of cell lysates were transferred to 96-well flat bottom luminometer plates, followed by the addition of luciferase substrate. The luciferase activity was measured in an Ultra 384 luminometer (Tecan). The percent inhibitory was calculated using the following formula: [1 − (E − N)/(P − N)] × 100. “E” represents the luciferase value in the experiment group. “N” represents the luciferase value in the negative control group, to which no virus was added. “P” represents the luciferase value in the positive control group, to which no inhibitor was added. The concentration for 50% inhibition (IC_50_) of cell fusion by an inhibitor was calculated using Calsusyn as described elsewhere [20].

### 2.4. Multiple-Cycle Viral Infection

The inhibitory activity of inhibitors against infection by CXCR4-tropic HIV-1 in MT-2 cells was determined as previously described [21]. Briefly, 1 × 10^4^ MT-2 cells were infected with HIV-1 at 100 TCID_50_ in 200 μL culture medium in the presence or absence of an inhibitor at graded concentrations at 37 °C overnight. Then the culture supernatants were changed with fresh medium. On the fourth day post-infection, 100 μL of culture supernatant was collected from each well and mixed with equal volumes of 5% Triton X-100. The virus lysates were determined for p24 antigen by ELISA. In brief, wells of 96-well polystyrene plates were coated with 5 μg/mL anti-p24 mAb (183-12H-5C) in carbonate-bicarbonate buffer (pH 9.6) at 4 °C overnight, followed by washing with PBS-T buffer and blocking with PBS containing 1% dry fat-free milk. Virus lysates were added to the wells and incubated at 37 °C for 1 h. After extensive washes, anti-p24 mAb (ab63193), biotin-labeled anti-rabbit IgG, streptavidin-labeled horseradish peroxidase (SA-HRP) and 3,3’,5,5’-tetramethylbenzidine (TMB) (Sigma) were added sequentially. Reactions were terminated by addition of 1N H_2_SO_4_. Absorbance at 450 nm was recorded in a microplate reader (Tecan).

The inhibitory activity of inhibitors on infection by HIV-1 in PBMC was determined as previously described. The PHA-stimulated PBMC were infected with HIV-1 in the absence or presence of a inhibitors at graded concentrations. On the fourth day post-infection, 100 μL of culture supernatant was collected from each well and mixed with equal volumes of 5% Triton X-100. The virus lysates were determined for p24 antigen by ELISA as described above. The percent of inhibitory and the calculation of IC_50_ were performed as described above.

### 2.5. Time-of-Addition Assay

HIV-1 IIIB at 100 TCID_50_ was incubated with 1 × 10^5^/mL MT-2 cells for 0, 0.5, 1, 2, 4, 6 and 8 h at 37 °C before the addition of SDF-1/54 (800 nM), AMD3100 (400 nM) and AZT (100 nM), respectively. After 24 h post-infection, the culture supernatants were replaced with fresh medium. On the fourth day post-infection, the culture supernatants were collected for measuring p24 antigen as described above.

### 2.6. HIV-1 Env-Mediated Cell–Cell Fusion

The effects of an inhibitor on HIV-1 Env-mediated viral fusion/entry was measured using a non-infectious cell–cell fusion assay, in which MT-2 cells and the CHO-WT cells that are engineered to express HIV-1 Env as target and effector cells, were used respectively [22,23]. Briefly, 1 × 10^5^ MT-2 cells were pre-treated with SDF-1/54 or other inhibitors in 96-well plate at 37 °C for 15 min. Then 1 × 10^5^ CHO-WT cells were added and cells were incubated at 37 °C for 48 h. Syncytia were counted under an inverted microscope (Axio Observer, Germany). The final concentration of each inhibnitor in the culture was 800 nM for SDF-1/54, 400 nM for AMD3100 and 100 nM for ADS-J1. The percent inhibition of cell fusion and the IC_50_ values were calculated using CalcuSyn software [20].

A dye transfer assay was performed using MT-2 cells as the target cells and HIV-1 IIIB chronically infected H9 cells (H9/HIV-1 IIIB) as the effector cells, as previously described [22,24]. Briefly, H9/HIV-1 IIIB cells (2 × 10^5^/mL) were labeled with calein-AM (1mM), a fluorescent reagent, at 37 °C for 30 min and then incubated with MT-2 cells (1 × 10^6^/mL) at 37 °C for 2 h in the presence or absence of inhibitor. The calcein-AM-labeled H9/HIV-1 IIIB cells, both fused and unfused with MT-2 cells, were counted under an inverted fluorescent microscope as described above. The fused cell is much larger (at least 2-fold) than the unfused cell and the intensity of fluorescence in the fused cell is much weaker than in the unfused cell because of the diffusion of fluorescence from one cell to two or more cells. The average percentage of cell fusion was calculated by the following formula: fused cells/(fused + unfused cells) × 100%. The percentage of inhibition of cell fusion by inhibitors was calculated by the formula: [1 − (%fusion in experiment − %fusion in negative control)/(% fusion in positive control − % fusion in negative control)] × 100%. The positive control or negative controls were wells that were added with H9/HIV-1 IIIB cells or H9 cells in the absence of antiviral agents, respectively. The IC_50_ values were calculated using CalcuSyn software.

### 2.7. CXCR4 Internalization Assay

For analysis of the amounts of CXCR4 on the cell surface, MT-2 cells were plated into each well of a 24-well tissue culture plate and treated with AMD3100 (50 nM) or SDF-1/54 (50 nM) for 2 h. Cells were harvested and incubated with PE-conjugated mouse anti-human CXCR4 antibody (Clone: 12G5, R&D) at 37 °C for 30 min. Cells were analyzed using flow cytometry following two washes with PBS. The control was the cells incubated with isotype control antibody.

### 2.8. Drug Combination Studies

The assays for evaluating the HIV-1 NL4-3 and IIIB infection in TZM-bl cells were used to test the synergistic antiviral effect of the SDF-1/54 with other FDA-approved antiviral (ARV) agents. Drugs tested in combination with SDF-1/54 included NRTIs AZT and tenofovir; the NNRTIs nevirapine and TMC120; and the integrase inhibitor raltegravir [25]. The anti-HIV-1 activity of SDF-1/54 and each ARV was assessed individually, or in combination, at a fixed molar ratio for the greatest synergism over a range of serial dilutions. The analysis was executed progressively by calculating IC_50_ or IC_90_ values based on the inhibition curves of a single drug or two drugs tested in combination. Then, the combination index was determined by calculating the median effect equation with the Calcusyn program to assess the synergistic effect of combinations [26]. A combination index of <1 indicates synergism. Combination index values are interpreted as follows: <0.1, very strong synergism; 0.1–0.3, strong synergism; 0.3–0.7, synergism; 0.7–0.85, moderate synergism; and 0.85–0.90, slight synergism. A combination index of 1, or close to 1, indicates additive effects and a combination index of >1 indicates antagonism. Dose reductions were calculated as the ratio between the IC_50_ of the drug used alone and in combination.

### 2.9. Cytotoxicity Assay

Cytotoxicity of SDF-1/54 toward the virus target cells (CEMx174 5.25M7 cells, Jurkat cells, TZM-bl cells, MT-2 cells and PBMCs) and vaginal epithelial cells (Hela cells, Siha cells, HEC-1-A cells, HEC-1-B cells and Ect/E6E7 cells) was evaluated by performing a 3-[4–dimethylthiazol-2-yl]-2, 5-diphenyltetrazolium bromide (MTT) cellular reduction assay. In brief, cells were seeded at a density of 20,000 per well in 96-well plates overnight for attachment and then incubated for 72 h with SDF-1/54 at graded concentrations. Next, MTT was added to each well and the cells were further incubated for 4 h. The colored formazan product was determined at 570 nm in a multi-well plate reader (Tecan). The 50% cytotoxicity concentrations (CC_50_) were calculated using CalcuSyn software.

### 2.10. Determination of the Inhibitory Activity of SDF-1/54 in PBS, SE-F, VFS and Human Serum Albumin (HSA) Respectively

Semen fluid (SE-F) was prepared by centrifuging the fresh semen (SE) at 500 g for 30 min to remove spermatozoa [27]. Vaginal fluid simulant (VFS) was prepared as described elsewhere [28]. SDF-1/54 was incubated with PBS, SE-F, VFS and HSA (45 mg/mL) at 37 °C, respectively. At different time points, samples were withdrawn for either visualization of the protein band by SDS-PAGE or determination of the remaining anti-viral activities in TZM-bl cells. As well, SDF-1/54 at graded concentration was incubated with PBS, SE-F, VFS and HSA (45 mg/mL) at 37 °C for 96 h, then, the samples were used to test their remaining anti-viral activities in TZM-bl cells. To avoid the interference of other protein or the toxic effects of SE-F, VFS, the mixtures were diluted with PBS or culture medium 50 times before SDS-PAGE analysis and testing the anti-viral activity as described above.

## 3. Results

### 3.1. Antiviral Potency of SDF-1/54

**Figure 1:**
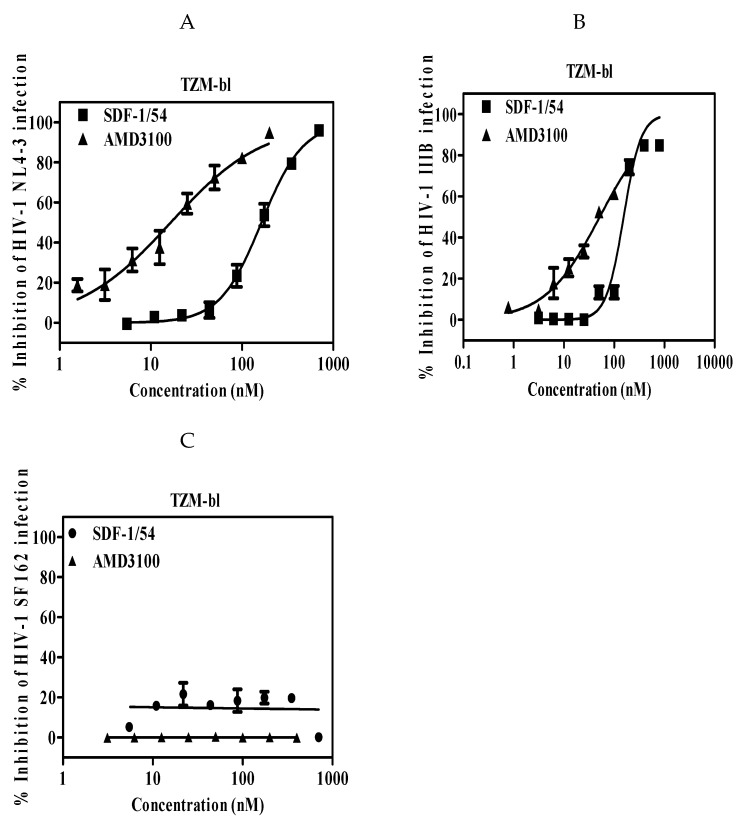
Antiviral potency of SDF-1/54 in a single-cycle viral infection. SDF-1/54-mediated inhibition of infection by infectious clone HIV-1 NL4-3 (**A**), laboratory-adapted HIV-1 IIIB (**B**) and HIV-1 SF162 (**C**) of TZM-bl cells. TZM-bl cells were pre-incubated with graded concentrations of SDF-1/54 for 15 min at 37 °C. Next, HIV-1 was added to the mixture. At 48 h post-infection, luciferase activity was detected. Average values (± SD) from three independent experiments are shown.

**Figure 2:**
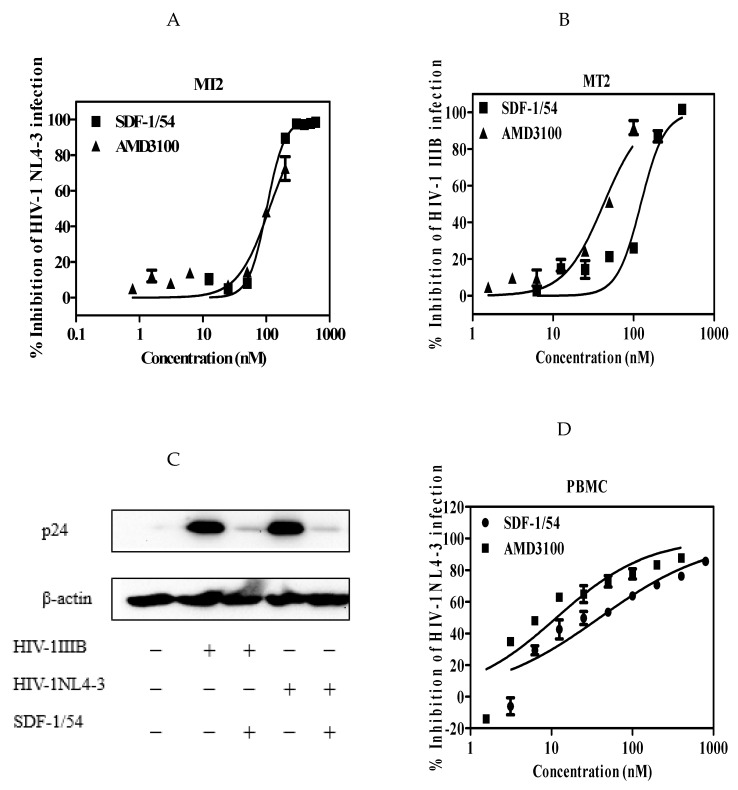
Antiviral potency of SDF-1/54 in multiple-cycle viral infection assays. SDF-1/54-mediated inhibition of infection by infectious clone HIV-1 NL4-3 (**A**) and laboratory-adapted HIV-1 IIIB (**B**) of MT-2 cells. MT-2 cells were pre-incubated with graded concentrations of inhibitor for 15 min at 37 °C. Next, mixtures were mixed with viruses. The culture medium was refreshed after overnight incubation. At 96 h post-infection, supernatant was collected and lyzed for p24 determination by ELISA and Western blotting (**C**). (**D**) SDF-1/54-mediated inhibition of infection by infectious clone HIV-1 NL4-3 in PBMC. Average values (±SD) from three independent experiments are shown.

The potency and efficacy of SDF-1/54 against CXCR4 tropic HIV-1 strains were determined in single-cycle infection assays and multiple-cycle infection assays, respectively. Firstly, we used a reporter cell line, TZM-bl cell, which is genetically engineered to stably express high levels of CD4 and HIV-1 co-receptors CCR5 and CXCR4 and also to contain the luciferase and β-galactosidase gene under the control of the HIV-1 long terminal repeat promoter. It is commonly used to measure HIV-1 infection level with advantages of being fast and cost-effective [29]. HIV-1 infectious clone NL4-3 and laboratory-adapted HIV-1 IIIB were used. The anti-viral activities of SDF-1/54 against the tested two HIV-1 strains in TZM-bl cells were similar, with IC_50_ values of 164.01 ± 14.40 nM for HIV-1 NL4-3 (Figure 1A and Table 1) and 156.25 ± 23.05 nM for HIV-1 IIIB (Figure 1B and Table 1). In contrast, SDF-1/54 was not active against the CCR5 HIV-1 strain SF162 at the concentration up to 700 nM (Figure 1C), thereby demonstrating selectivity of SDF-1/54 for X4-tropic virus. Then, we used MT-2 cell as target cells, in which newly generated virus is released and infects target cells. SDF-1/54 proved active against the X4 HIV-1 strains NL4-3 (Figure 2A,C) and IIIB (Figure 2B,C) with IC_50_ being 102.84 ± 15.56 nM and 113.37 ± 23.23 nM (Table 2). We also tested the inhibitory activities of SDF-1/54 on HIV-1 NL4-3 infection in PBMC. The IC_50_ of SDF-1/54 was 41.81 ± 13.73 nM and AMD3100 was 12.44 ± 4.61 nM (Figure 2D and Table 2).

**Figure 3:**
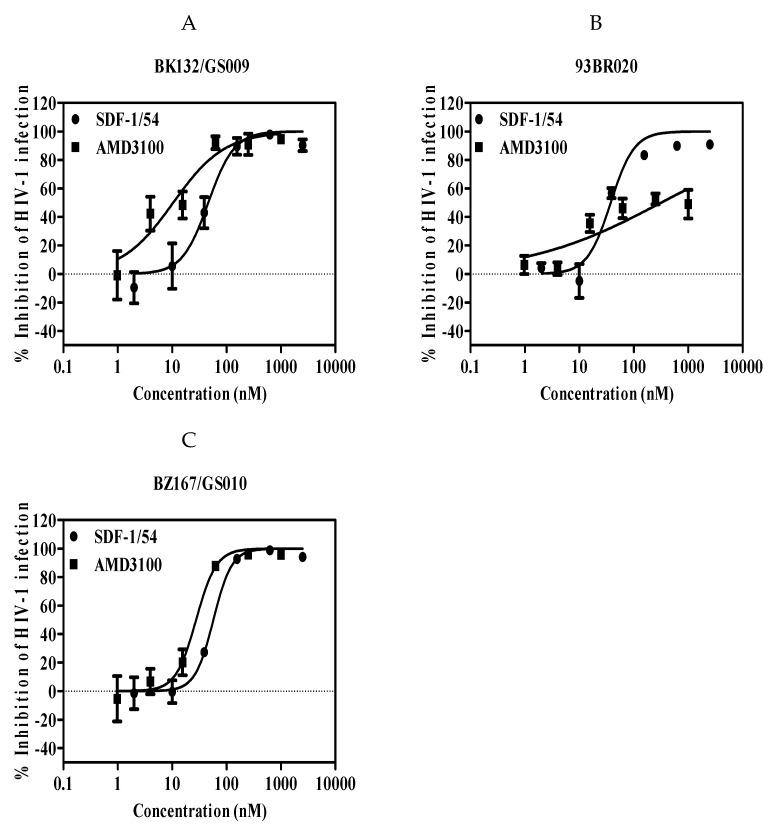
SDF-1/54-mediated inhibition of infection by primary HIV-1 isolates of TZM-bl cells. Inhibition of the primary X4-tropic HIV-1 isolates included BK132/GS009 (**A**), 93BR020 (**B**) and BZ167/GS010 (**C**). TZM-bl cells were pre-incubated with graded concentrations of SDF-1/54 for 15 min at 37 °C. Next, primary HIV-1 isolates were added to the mixture. At 48 h post-infection, luciferase activity was detected. Average values (±SD) from three independent experiments are shown.

Next, we also investigated the effects of SDF-1/54 on inhibiting infection by primary HIV-1 isolates of TZM-bl cells. We showed that SDF-1/54 inhibited infection of TZM-bl cells by all primary X4 monotropic HIV-1 isolates tested (Figure 3 and Table 3). The IC_50_ values for inhibition of primary HIV-1 infection in TZM-bl ranging from about 55 to 92 nM. SDF-1/54 could also inhibit dual-tropic primary HIV-1 isolates infection in TZM-bl cells.

**Figure 4:**
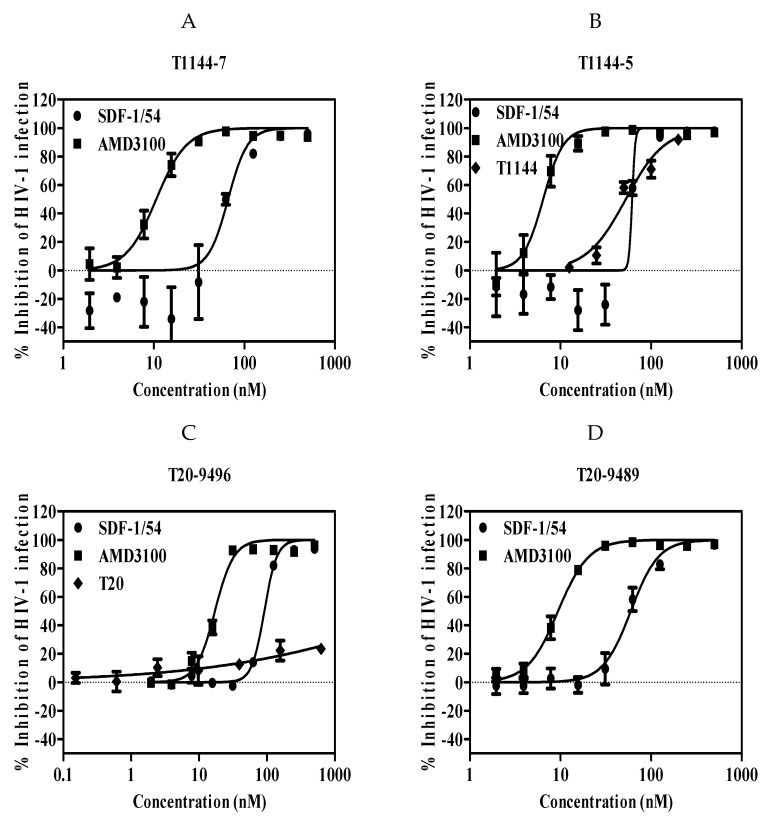
SDF-1/54 is effective against HIV-1 drug-resistant strains. SDF-1/54-mediated inhibition of infection by drug-resistant HIV-1 of TZM-bl cells which included viral strains resistant to T1144 (**A**,**B**) and T20 (**C**,**D**). TZM-bl cells were pre-incubated with graded concentrations of SDF-1/54 for 15 min at 37 °C. Then, drug-resistant HIV-1 was added to the mixture. At 48 h post-infection, luciferase activity was detected. Average values (±SD) from three independent experiments are shown.

We further determined whether SDF-1/54 was effective against HIV-1 drug-resistant strains. Viral strains resistant to T20, an HIV-1 entry/fusion inhibitor and T1144 were used. Results showed that the IC_50_ values for inhibition of these drug-resistant HIV-1 infection in TZM-bl ranged from about 96 to 128 nM (Figure 4 and Table 4).

### 3.2. SDF-1/54 Inhibits HIV-1 Entry by Time-of-Addition Assay

**Figure 5:**
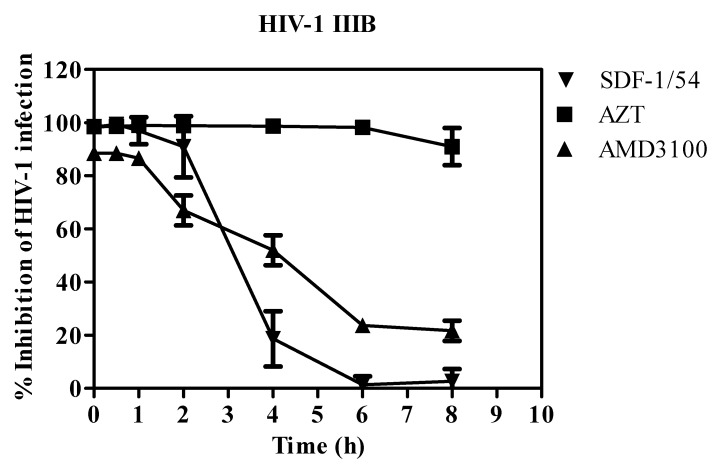
Inhibition of HIV-1 entry by SDF-1/54 as determined by the time-of-addition assay. SDF-1/54 was added to MT-2 cells at different intervals after infection with HIV-1 IIIB. AZT and AMD3100 were included as controls. The final concentration was 800 nM for SDF-1/54, 100 nM for AZT and 400 nM for AMD3100. Averages (±SD) from three independent experiments are shown.

Because SDF-1/54 targets the co-receptor CXCR4, which is essential for viral entry, we validated its role as an HIV-1 entry inhibitor by a time-of-addition assay in which we studied the inhibitory activity of SDF-1/54 against CXCR4-tropic HIV-1 IIIB when it was added to cells at different intervals post-infection. Figure 5 shows the timing effect of SDF-1/54 on its anti-HIV-1 activity compared to other ARVs. The nucleoside reverse transcriptase inhibitor AZT exhibited similar anti-HIV-1 activities against HIV-1 IIIB when it was added to cells 2 h or 8 h post-infection, while the HIV-1 entry inhibitor AMD3100 exhibited significant decreased inhibitory activities when it was added more than 2 h post-infection. SDF-1/54 showed inhibitory profiles similar to those of HIV-1 entry inhibitors, indicating that SDF-1/54 is also an HIV-1 entry inhibitor.

### 3.3. SDF-1/54 Inhibits HIV-1 Entry by Blocking Membrane Fusion

**Figure 6:**
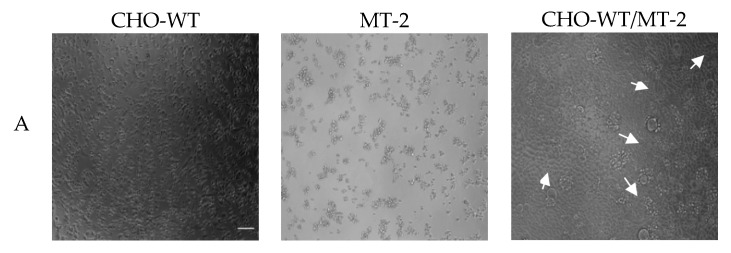

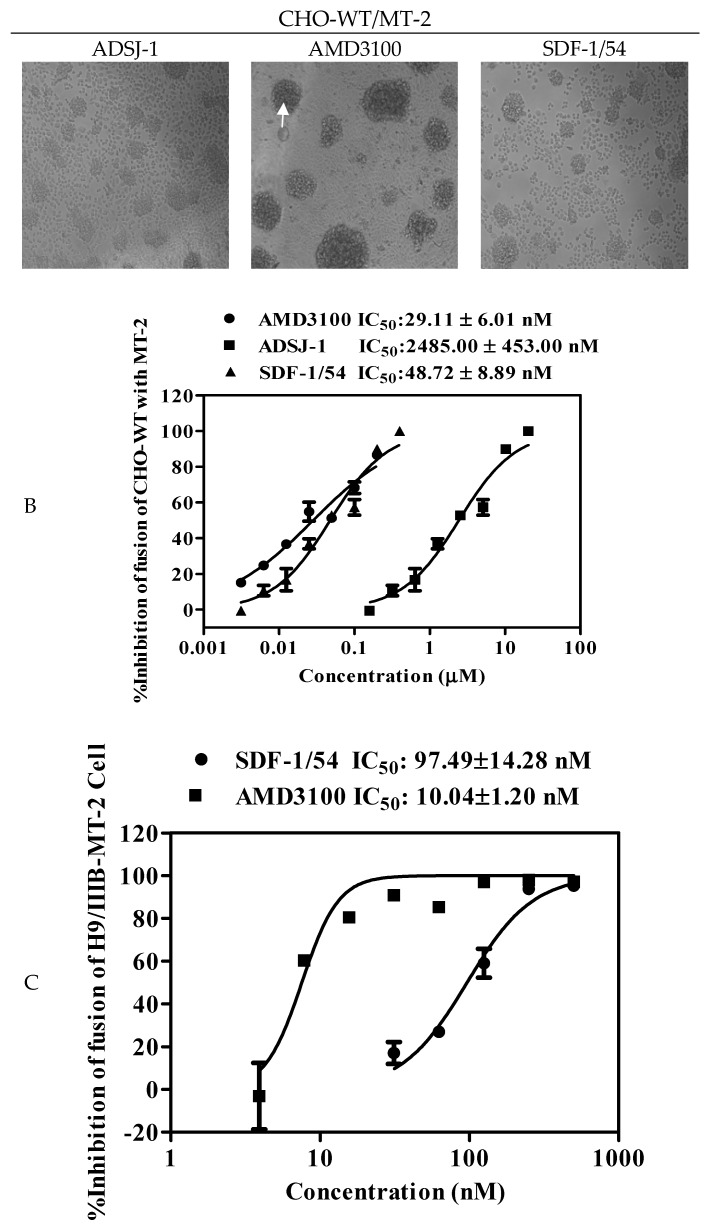
The inhibitory activity of SDF-1/54 against HIV-1-mediated cell–cell fusion. Effects of SDF-1/54 on the formation of syncytium with CHO-WT and MT-2 cells (**A**). Among them, the white arrow in the figure indicates the fusion bubble of cell fusion. Scale bar = 100 μm. (**B**) Dose-response curves and IC_50_ values of SDF-1/54 inhibiting cell–cell fusion. ADS-J1 was chosen as a positive control. (**C**) Cell–cell fusion between calcein-labeled H9/HIV-1 IIIB and MT-2 cells. Data are presented as mean ± SD.

Viral envelope glycoprotein (Env)-mediated membrane fusion is a critical step for HIV-1 entry into target cells. Therefore, it is vital to determine whether SDF-1/54 can inhibit HIV-1 Env-mediated cell–cell fusion. We used noninfectious CHO-WT cells expressing HIV-1 Env as the effector cells and MT-2 cells expressing CD4 and CXCR4 as the target cells to determine the inhibitory activity of SDF-1/54 against HIV-1 Env-mediated cell–cell fusion. The formation of syncytium with CHO-WT and MT-2 cells was imaged in Figure 6A. As shown in Figure 6B, SDF-1/54 inhibited cell–cell fusion in a dose-dependent manner, with an IC_50_ of about 48.72 ± 8.89 nM, more effective than the positive control ADS-J1 (IC_50_, 2485.00 ± 453.00 nM).

In addition, we investigated the inhibitory effects of SDF-1/54 on membrane fusion by an infectious cell fusion assay, in which the fluorescent dye is transferred from HIV-1-infected cells (H9/HIV-1 IIIB) to uninfected cells (MT-2) when the cells fused. As shown in Figure 6C, SDF-1/54 could effectively inhibit HIV-1 Env-mediated cell–cell fusion, resulting in the reduction of dye transfer in a dose-dependent manner, with IC_50_ of about 97.49 nM. Results showed that SDF-1/54 inhibited both non-infectious and infectious HIV-1 Env-mediated cell–cell fusion.

### 3.4. SDF-1/54 Inhibits HIV-1 Entry by Inducing CXCR4 Internalization

**Figure 7:**
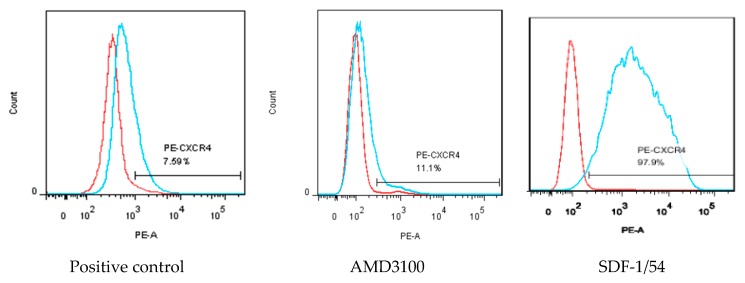
Capability of SDF-1/54 inducing CXCR4 internalization. MT-2 cells were treated with AMD3100 and SDF-1/54 for 2 h at 50 nM and CXCR4 on the cell surface was detected indirectly by PE-labeled CXCR4 antibody using flow cytometry. The level of CXCR4 on the cell surface is shown as mean fluorescence density, as described in Materials and Methods. MT-2 cells without any treatment were used as a control.

MT-2 cells were used to investigate whether SDF-1/54 inhibited HIV-1 entry by inducing CXCR4 internalization. The amount of CXCR4 on the cell surface was measured by flow cytometry after treating the cells with 50 nM of AMD3100 or SDF-1/54 for 2 h. The results showed that the amount of CXCR4 on the surface of AMD3100 and SDF-1/54-treated cells was significantly reduced compared to the untreated cells, indicating that SDF-1/54 retained the ability to bind with and internalize CXCR4. SDF-1/54 inhibits HIV-1 entry by inducing a CXCR4 internalization similar to AMD3100 (Figure 7).

### 3.5. SDF-1/54 Showed Limited in Vitro Cytotoxicity to HIV-1 Target Cells and Reproductive Tract Epithelial Cells

The efficacy of a drug depends on the balance between its specific activity and its safety. Therefore, we evaluated the cytotoxicity of SDF-1/54 to the target cells that were used for assessing anti-HIV-1 activity of SDF-1/54, including CEMx174.525 M7 cells, MT-2 cells, TZM-bl cells, Jurkat cells, PBMCs and the vaginal and cervical epithelial cells lines, including Siha cells, Hela cells, HEC-1-A cells, HEC-1-B cells and Ect/E6E7 cells. SDF-1/54 displayed little in vitro cytotoxicity to these cells even at the concentration of 3000 nM, which is more than 20 times its 50% inhibitory concentration (IC_50_) against HIV-1 NL4-3 infection in TZM-bl cells (Table 5 and Figure S1).

### 3.6. Combinations of SDF-1/54 with ARVs Display Synergistic Effects Against HIV-1 Infection

The above studies have shown that SDF-1/54 could potently inhibit CXCR4-tropic HIV-1 infection by blocking HIV-1 entry into host cells. Next, we investigated the potential cooperative effects of SDF-1/54 combined with various ARV drugs on infection against HIV-1 IIIB and NL4-3 strains in TZM-bl cells. The ARV drugs used included reverse transcriptase inhibitors (NRTIs) (zidovudine (AZT) and tenofovir (TNF)), non-nucleoside reverse transcriptase inhibitors (NNRTIs) (nevirapine (NVP) and dapivirine (TMC120)) and an integrase inhibitor (raltegravir RAL). The data in Table 6 and Table 7, Figures S2 and S3 show that combining SDF-1/54 with any of the above ARV drugs produced strong synergistic and complementary effects against infection by HIV-1 IIIB and NL4-3 in TZM-bl respectively. The observed 50% competitive index (CI_50_) values ranged from 0.269–0.683 and the IC_50_ values of the individual drugs in each combination were reduced by approximately 2.04–51.51-fold. The strongest synergism was observed when SDF-1/54 was combined with the raltegravir, with value of CI_50_—being 0.269. Dose reductions in the IC_50_ values of SDF-1/54 and raltegravir were approximately 4–51-fold against HIV-1 IIIB, respectively. This combination is expected to exert synergistic antiviral effects and to help prevent the development of drug resistance during the prevention and treatment of HIV-1 infection.

### 3.7. SDF-1/54 Remained Stable and Potent Anti-HIV-1 Activity in the Presence of Human Body Fluids

**Figure 8:**
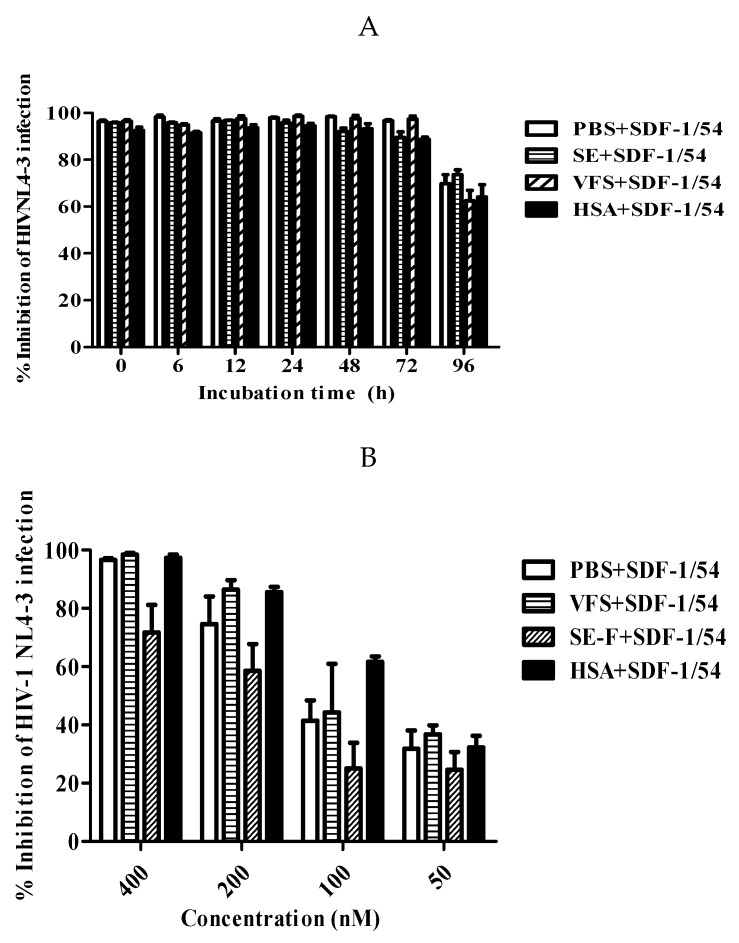
The effects of human SE-F, VFS and HSA on the anti-HIV-1 stability and activity of SDF-1/54. SDF-1/54 was incubated with PBS, VFS, SE-F and HSA (45mg/mL) for various time period. The remaining anti-viral activities of SDF-1/54 were tested (**A**). In addition, different concentrations of SDF-1/54 maintained antiviral activity in the presence or absence of SE, VFS and HSA (**B**), which were determined using p24 ELISA assay. Averages (± SD) from three independent experiments are shown.

HIV-1 infection or transmission occurs in the presence of human body fluids, such as human blood, seminal and vaginal fluids, which may have negative effects on the efficacy of an anti-viral agent [30,31]. Therefore, it is necessary to determine the potential effect of these human body fluids on the anti-HIV activity of SDF-1/54. Blood usually contains abundant HSA; we tested the stability and inhibitory activities of SDF-1/54 in the presence SE-F, VFS and HSA at physiological plasma protein levels. As shown in Figure 8 and Figure S4, HSA, SE-F and VFS did not show significant effects on the stability and inhibitory effects of SDF-1/54 over an incubation period of 96 h, compared to the PBS control. In addition, SDF-1/54 remained with unaltered antiviral activity in the presence of SE-F, VFS and HSA, even 96 h after its incubation with different body fluids (Figure 8B and Figure S4). The results suggest that SDF-1/54 is stable and that SE-F, VFS and HSA have no negative effect on the application of SDF-1/54 as an anti-HIV-1 agent.

## 4. Discussion

In theory, HIV-1 is inhibited from entering any part of the cell which can inhibit HIV-1 infection and thus achieve the goal of treating AIDS. In order to block HIV-1 infection from the beginning, there has been a growing interest in developing an entry/fusion inhibitor as therapeutic intervention. Entry inhibitor might act rapidly to block HIV-1 before the virus establishes infection in target cells. In 1996, the role of the co-receptor CXCR4 in the process of entry of HIV-1 was originally discovered and SDF-1 inhibited the fusion and replication of HIV-1 in cells. Further studies have shown that endogenous SDF-1 can competitively bind to CXCR4 on T cell membrane, thereby, inhibiting the entry of X4-tropic HIV-1 into T cells and protecting T cells from infection [7,9]. Chemokine-mediated inhibition of HIV-1 entry seems to be the result of the combination of three mechanisms: (i) steric blocking of the interaction between gp120 and co-receptors; (ii) ligand-mediated receptor internalization, which reduces the availability for use by gp120; and (iii) interference with receptor recycling. So far, several findings have shown that chemokine receptor internalization is the main mechanism by which chemokines prevent host cells from infecting HIV-1 [32]. However, CXCR4 is widely expressed in various hematogenous cells. It participates in many biological activities through signal transduction after binding to SDF-1 [33]. For example, SDF-1/CXCR4 plays roles in immune [34], hematopoietic [35], brain development [36], angiogenesis [37,38], HIV-1 infection [39], autoimmune diseases [34,40], cancer [41,42], inflammation [43] and other pathological processes [44]. Due to the multifunctional nature of SDF-1, the risk of SDF-1 inducing inflammatory side effects or interfering with the physiological nature of homeostasis chemokine system potentially limits its therapeutic application. When SDF-1 is employed to be an anti-HIV-1 agent, there will be a whole host of side effects [9]. Moreover, it has been verified that CXCR4 was internalized when SDF-1 interacts with CXCR4. After conducting a series of physiological activities, CXCR4 returns to the cell surface [45,46]. Therefore, a good inhibitor targeting CXCR4 should be able to recognize and specifically bind to CXCR4 without activating the CXCR4-related signaling pathway and affecting its normal biological function.

Based on the study of structure–activity relationship of SDF-1/CXCR4, our team deleted the α-helix of the C-terminal functional region of SDF-1 and obtained an SDF-1 mutant, SDF-1/54. Our previous studies showed that SDF-1/54 which has a defective C-terminal a-helix, a normal N-terminus and a normal central β-strand scaffold structure, retains normal binding affinity to CXCR4 and normal induction of CXCR4 internalization, but fails to activate CXCR4-mediated intracellular calcium influx, extracellular signal-regulated kinase (ERK) phosphorylation and chemotaxis. Therefore, SDF-1/54 might be seen as an optimal CXCR4 antagonist. This study suggests that SDF-1/54 blocks the function of CXCR4 as an HIV-1 co-receptor by interacting with the CXC-chemokine receptor CXCR4. Since HIV-1 inhibition by the SDF-1 analogs correlated well with their affinities for CXCR4, the inhibitory effect of SDF-1/54, which retains the co-receptor binding site on HIV-1, is not significantly different from that of SDF-1. This is the advantage of SDF-1/54 over other SDF-1 mutants that alter the N-terminus [7]. Furthermore, the lack of activity of SDF-1/54 on inhibiting CCR5-tropic viruses indicates that it is specific to CXCR4 and not a general inhibitor of HIV-1 entry. In addition, SDF-1/54 inhibits env-mediated membrane fusion in the absence of other HIV-1 proteins, suggesting that the expression of gene products other than the envelope glycoprotein is not necessary for this protein to exert antiviral activity.

SDF-1/54 displayed antiviral activity against CXCR4-tropic HIV-1 with low cytotoxicity. SDF-1/54 is effective in inhibiting infection of primary X4 viruses with distinct genotypes and phenotypes. Interestingly, SDF-1/54 was shown to be effective against the HIV-1 variants that are resistant to T20 and T1144, the first and next generation HIV-1 fusion inhibitors, suggesting that SDF-1/54 is capable of preventing HIV-1 transmissions that are resistant to the currently used antiretroviral therapeutics.

The antiviral potency of an ideal anti-viral agent should not be significantly decreased when it is in the presence of human body fluids, including human vaginal secretion, semen and blood. Our results showed that SDF-1/54 remained at a high level of antiviral potency against HIV-1 infection and that the presence of vaginal fluid simulant, seminal fluid and HSA had no significant effects on the antiviral potency of SDF-1/54, suggesting that SDF-1/54 is suitable for further development as an effective and safe anti-HIV agent.

SDF-1/54 may have an impact on its value as an anti-HIV-1 drug. HIV-1 uses multiple co-receptors to enter its target cells, so inhibiting only one, CXCR4, may not be sufficient for a significant anti-viral effect in vivo. However, blocking CXCR4 may prevent the emergence of the more virulent SI viruses that use this receptor, which could be beneficial. SDF-1/54 as an inhibitor of the interactions of HIV-1 with a co-receptor is a useful step towards the general development of HIV-1 entry antagonists. It may eventually be achievable to develop drug combinations which are able to block the use of multiple co-receptors. SDF-1/54 is derived from the deletion of natural protein. It is not only highly effective and of low toxicity and immunogenicity, but also easy to prepare by genetic engineering methods with high product yield and low cost. With its novel mechanism of action, the ability to inhibit drug-resistant strains and the good combination profile with other ARVs, SDF-1/54 could be a valuable member of such a therapeutic cocktail. However, it is only through carefully designed and conducted therapeutic trials that the clinical validity of these in vitro studies can be determined. Naiming Zhou confirmed that acting as CXCR4 antagonists and with much higher biological stability than L-counterparts, the D-peptides showed more potent activity in inhibiting the replication of CXCR4-tropic HIV-1 strains [47]. Whether D-SDF-1/54 in HIV-1 replication is more active than L-SDF-1/54 requires further study. The combination of SDF-1/54 with already existing anti-HIV-1 agents will certainly be a great step forward in anti-HIV-1 drug research.

## Figures and Tables

**Table 1:** The inhibitory activities of SDF-1/54 on infection by two HIV-1 strains in TZM-bl cells.

Virus Strain	IC_50_ (nM) ^a^ of	
	SDF-1/54	AMD3100
HIV-1 NL4-3	164.01 ± 14.40	16.97 ± 4.05
HIV-1 ⅢB	156.25 ± 23.05	52.16 ± 8.00

^a^ The measurements were from three independent experiments.

**Table 2:** The inhibitory activities of SDF-1/54 on infection by two HIV-1 strains in MT-2 cells and PBMC.

Virus Strain	Target Cells	IC_50_ (nM) ^a^ of	
		SDF-1/54	AMD3100
HIV-1 NL4-3	MT-2	102.84 ± 15.56	113.58 ± 18.23
HIV-1 IIIB	MT-2	113.37 ± 23.23	43.16 ± 6.75
HIV-1 NL4-3	PBMC	41.81 ± 13.73	12.44 ± 4.61

^a^ The measurements were from three independent experiments.

**Table 3:** The inhibitory activities of SDF-1/54 on infection by primary HIV-1 strains in TZM-bl.

Virus Strain	IC_50_ (nM) ^a^ of	
	SDF-1/54	AMD3100
BK132/GS009 (B, X4)	66.21 ± 27.00 nM	15.52 ± 6.02 nM
93BR020 (F,X4/R5)	91.63 ± 19.00 nM	>250.00 nM
BZ167/GS010 (B,X4)	54.90 ± 21.00 nM	32.10 ± 13.00 nM

^a^ The measurements were from three independent experiments.

**Table 4:** The inhibitory activities of SDF-1/54 and AMD3100 on infection by HIV-1 resistant strains.

Virus strain	IC_50_ (nM) ^a^ of			
	SDF-1/54	AMD3100	T20	T1144
T1144-7	95.19 ± 30.55	14.74 ± 9.36	ND ^b^	ND
T1144-5	84.09 ± 20.10	6.76 ± 9.76	ND	61.29 ± 12.83
T20-9496	127.29 ± 12.35	30.06 ± 1.74	2266.17 ± 521.34	ND
T20-9489	60.29 ± 8.43	9.45 ± 0.82	ND	ND

^a^ The measurements were from three independent experiments. ^b^ The measurements were not done.

**Table 5:** In vitro cytotoxicity of SDF-1/54.

Cell Lines	Cell Type	CC_50_
CEMx174 5.25M7 Jurkat	Infection target cells	>3000 nM
MT-2		
TZM-b1		
PBMCs		
Siha	Vaginal epithelial cells	
Hela		
HEC-1-A		
HEC-1-B		
Ect/E6E7		

**Table 6:** Combination index and dose reduction values for inhibition of HIV-1NL4-3 infection by combining SDF-1/54 with ARVs.

Drug Combination, % Inhibitory Conc. (Molar Ratio)	CI ^b^	Mean Value for ^a^:					
		SDF-1/54			ARVs		
		Conc. (nM)		Dose Reduction	Conc. (nM)		Dose Reduction
		Alone	Mixture		Alone	Mixture	
SDF-1/54:AZT (200:1)							
50	0.537	78.01	32.74	2.38	1.40	0.16	8.51
90	0.379	222.41	77.25	2.88	12.23	0.39	31.69
SDF-1/54:TNF (1:5)							
50	0.382	180.06	55.79	3.23	3854.03	278.94	13.82
90	0.722	413.89	279.57	1.48	29772.00	1397.84	21.30
SDF-1/54:RAL (60:1)							
50	0.643	198.15	74.88	2.65	1.37	1.25	1.10
90	0.420	277.09	105.78	2.62	25.39	4.62	5.50
SDF-1/54:NVP (10:1)							
50	0.452	78.01	32.74	2.38	33.34	2.85	11.68
90	0.288	222.41	77.25	2.88	146.74	5.55	26.43
SDF-1/54:TMC120 (30:1)							
50	0.683	118.37	57.93	2.04	9.97	1.93	5.16
90	0.401	344.40	90.30	3.81	21.67	3.01	7.20

^a^ The data shown are the means of three independent assays performed in triplicate; ^b^ The CI value reflects the nature of the interaction between drugs.

**Table 7:** Combination index and dose reduction values for inhibition of HIV-1 IIIB infection by combining SDF-1/54 with ARVs.

Drug Combination, % Inhibitory Conc. (Molar Ratio)	CI ^b^	Mean Value for ^a^:					
		SDF-1/54			ARVs		
		Conc. (nM)		Dose Reduction	Conc. (nM)		Dose Reduction
		Alone	Mixture		Alone	Mixture	
SDF-1/54:AZT (37.5:1)							
50	0.362	149.39	12.41	12.04	1.19	0.33	3.59
90	0.232	227.88	33.83	6.74	10.79	0.90	11.96
SDF-1/54:RAL (30:1)							
50	0.269	41.55	10.38	4.00	17.82	0.35	51.51
90	0.204	114.04	20.66	5.52	29.75	0.69	43.18
SDF-1/54:NVP (10:1)							
50	0.312	149.39	39.19	3.81	79.34	3.92	18.71
90	0.219	227.88	46.92	4.86	348.14	4.69	74.20
SDF-1/54:TMC120 (15:1)							
50	0.327	41.55	10.28	4.04	8.57	0.69	12.50
90	0.244	114.04	18.40	6.20	14.90	1.23	12.14

^a^ The data shown are the means of three independent assays performed in triplicate; ^b^ The CI value reflects the nature of the interaction between drugs.

## Supplementary Materials

The following are available online at https://www.mdpi.com/1999-4915/11/9/874/s1, Figure S1: In vitro cytotoxicity of SDF-1/54, Figure S2: Synergism achieved by combining SDF-1/54 and ARV drugs for inhibition of infection by HIV-1NL4-3; Figure S3: Synergism achieved by combining SDF-1/54 and ARV drugs for inhibition of infection by HIV-1 IIIB, Figure S4: The effects of PBS, human VFS, SE-F, and HSA on the stability of SDF-1/54.
