# Supplementary material for: A Novel CXCR4 Targeting Protein SDF-1/54 as an HIV-1 Entry Inhibitor
Source: Viruses. 2019 Sep 18;11(9):874. doi: 10.3390/v11090874 (PMC6783869; doi:10.3390/v11090874)
Supplement: Supplementary file 1 [file viruses-11-00874-s001.pdf]

SUPPLEMENTARY INFORMATION

A Novel CXCR4 Targeting Protein SDF-1/54 as an HIV-1 Entry Inhibitor

Suiyi Tan[1†], Wenjuan Li[1†], Zhaofeng Li[1†], Yujing Li[3], Jiangyan Luo[3], Liang zhentian Yu[3], Jie Yang[3], Mengjie Qiu[1], Hongyan Cheng[1], Wei Xu[2], Shibo Jiang[2], Lu Lu[2*] and Shuwen Liu[1*],Weifeng Ma[3*]

1. Guangdong Provincial Key Laboratory of New Drug Screening, School of Pharmaceutical Sciences, Southern Medical University, Guangzhou 510515, China;

2. Key Laboratory of Medical Molecular Virology of Ministries of Education & Health, Shanghai Medical College and Institute of Medical Microbiology, Fudan University, Shanghai 200032, PR China;

3 Department of Microbiology, School of Public Health, Southern Medical University, Guangzhou, 510515, China

† These authors contributed equally to this study.

* Correspondence:Tel:+862062789123, +862061648538, +862154237671; E-mail: maweifeng919@163.com; liusw@smu.edu.cn, lul@fudan.edu.cn

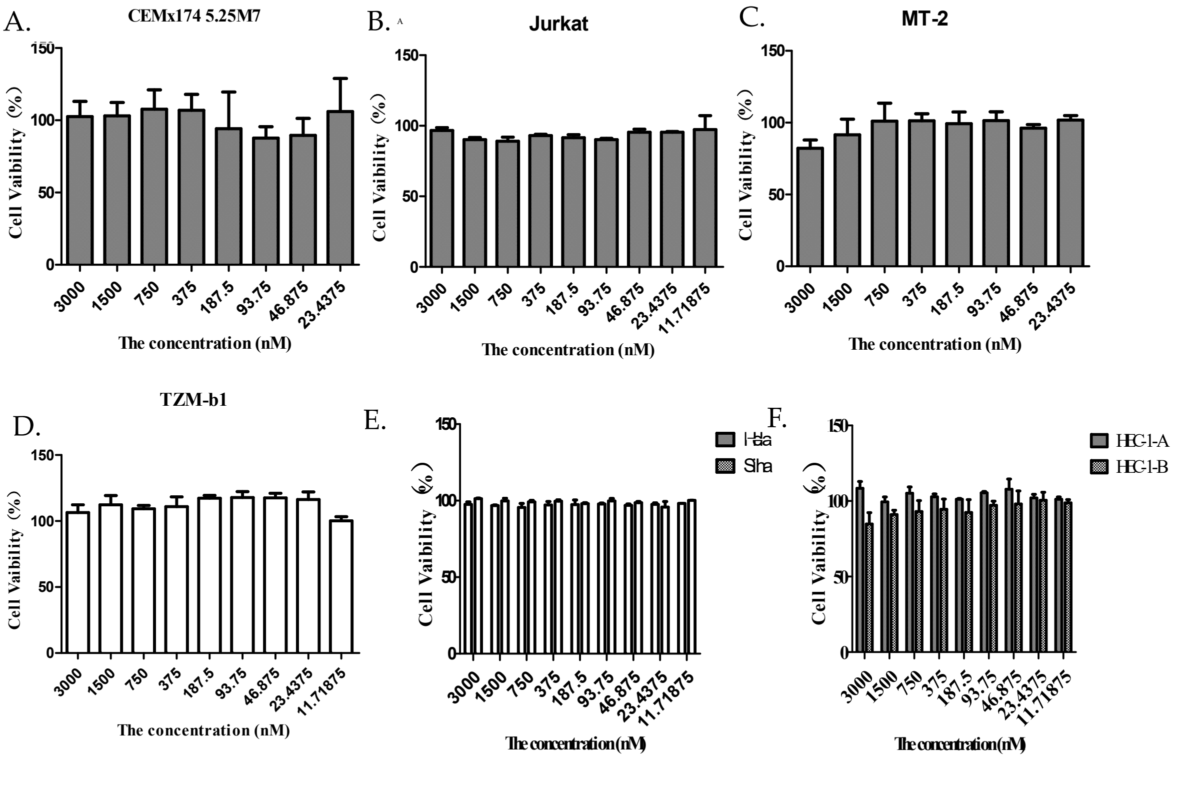

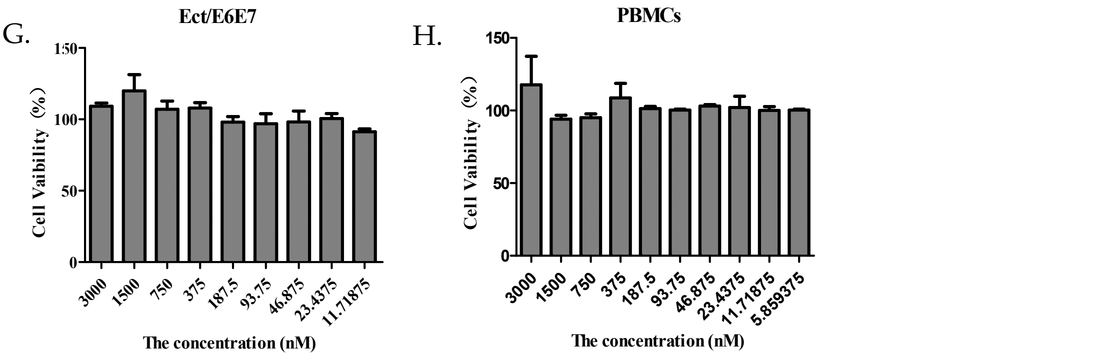

Figure S1:*In vitro* cytotoxicity of SDF-1/54.A. CEMx174 5.25M7 cells, B. Jurka cells, C. MT-2 cells, D. TZM-b1 cells, E. Hela cells and Siha cells, F. HEC-1-A cells and HEC-1-B cells, G. Ect/E6E7 cells, H. PBMC cells. The concentrations of SDF-1/54 double dilution from 3000 to 11.7 nM. The MTT assay was used to determine the cytotoxicity.

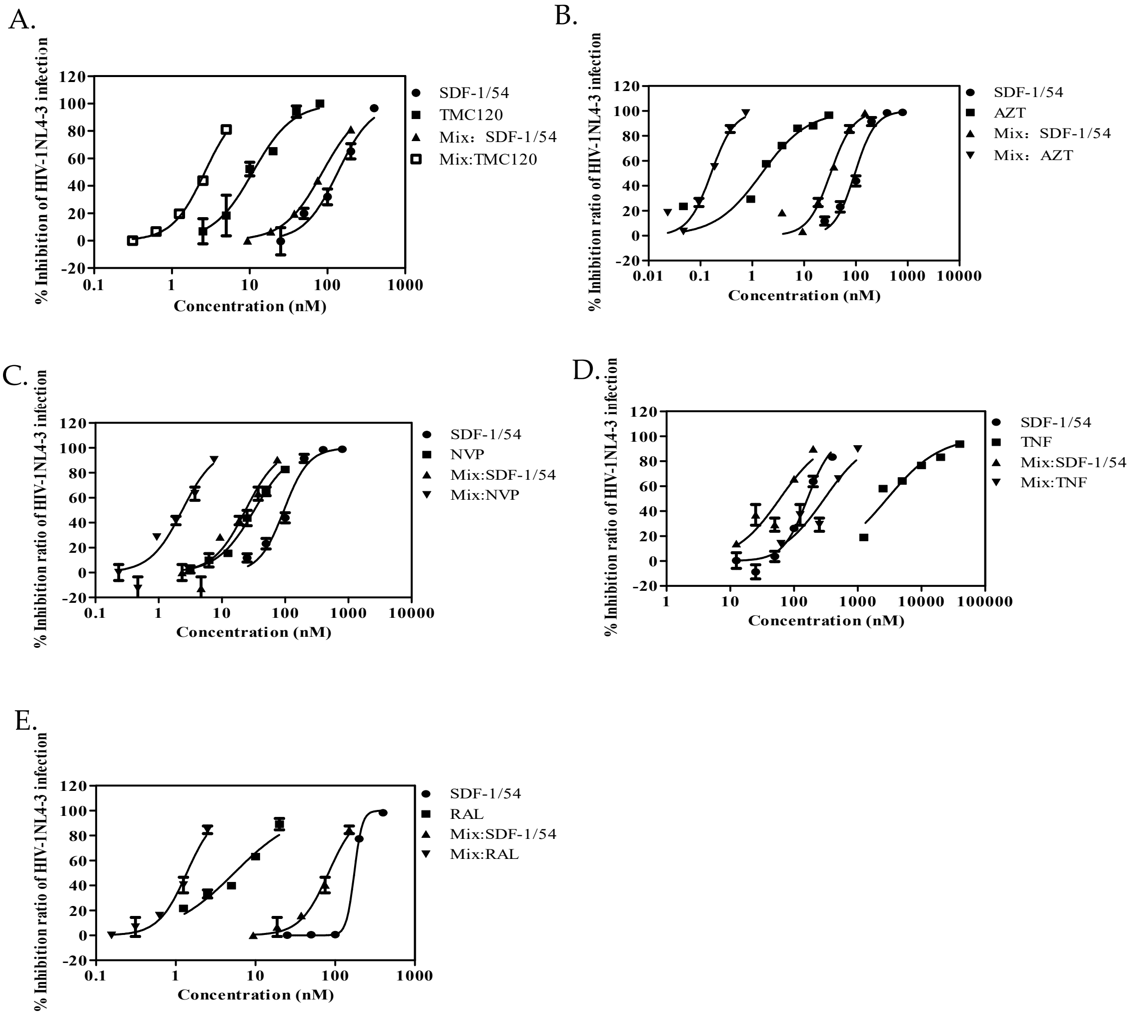

Figure S2: **Synergism achieved by combining SDF-1/54 and ARV drugs for inhibition of infection by HIV-1NL4-3.** Compounds were examined at fixed molar rations individually and in combination. A. SDF-1/54 and TMC120 (30:1); B. SDF-1/54 and AZT (200:1); C. SDF-1/54 and NVP (10:1), D.SDF-1/54 and TNF (1:5), E. SDF-1/54 and RAL (60:1).

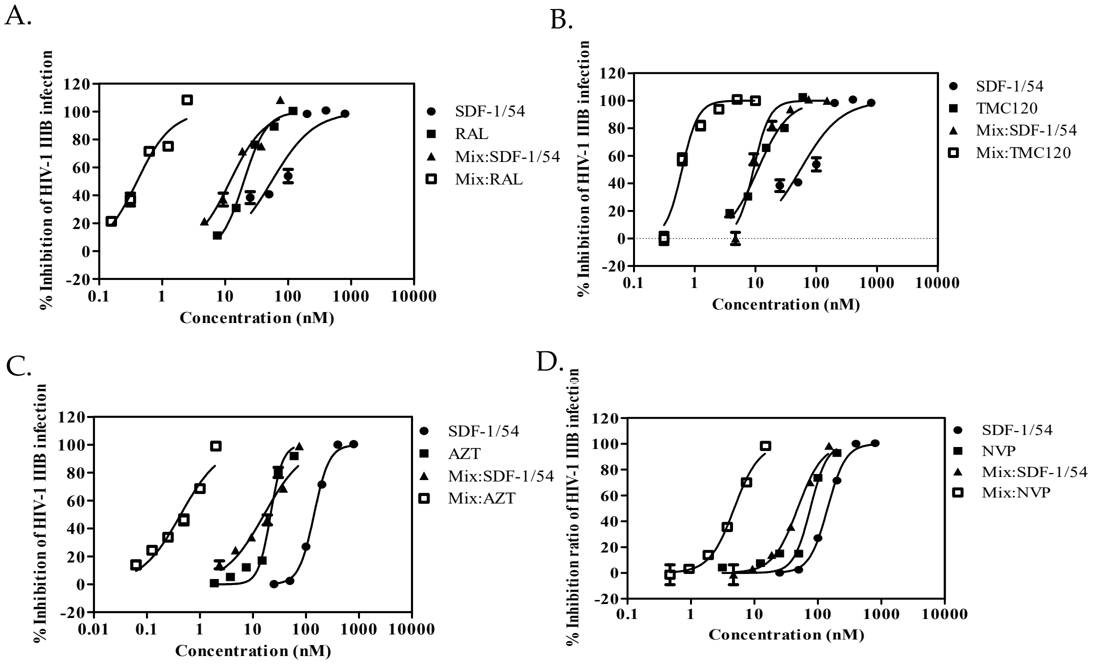

Figure S3: **Synergism achieved by combining SDF-1/54 and ARV drugs for inhibition of infection by HIV-1Ⅲ.** Compounds were examined at fixed molar rations individually and in combination. A. SDF-1/54and RAL (30:1); B. SDF-1/54 and TMC120 (15:1); C. SDF-1/54 and AZT (37.5:1) D.SDF-1/54 and NVP (10:1).

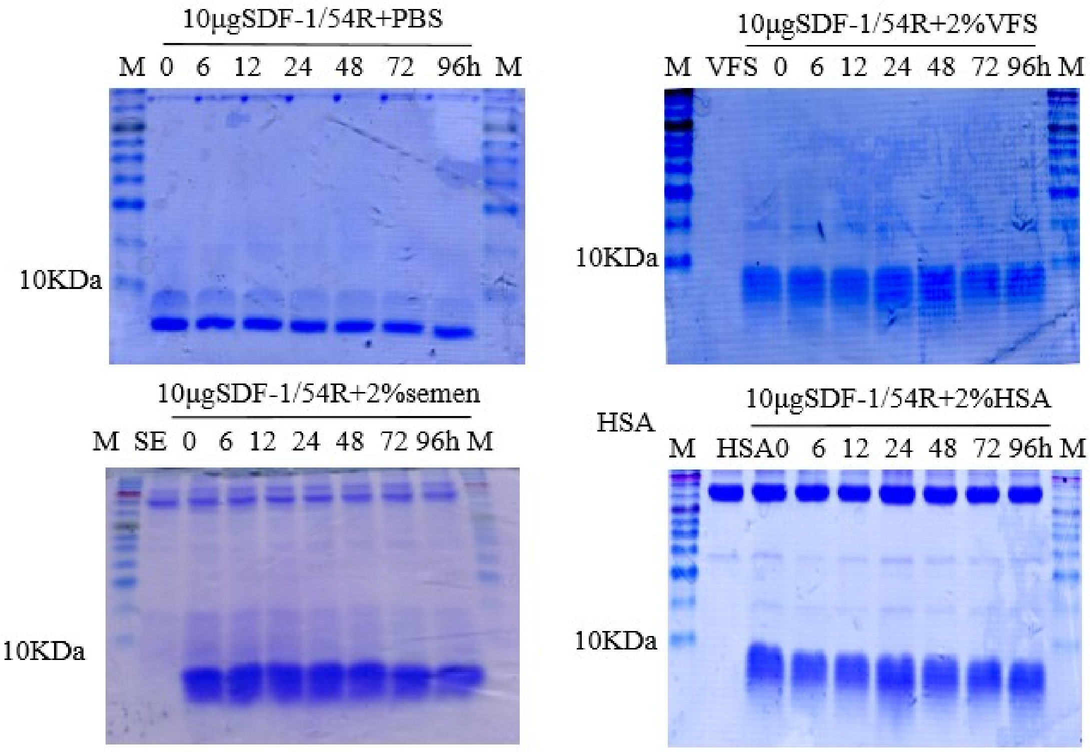

Figure S4: **The effects of PBS, human VSF, SE-F, and has on the stability of SDF-1/54.** SDF-1/54 was incubated with PBS, VSF, SE-F and HSA respectively for various time periods. The level of SDF-1/54 in the mixture was determined by SDS PAGE.
